# A chromosome-level reference genome facilitates the discovery of clubroot-resistant gene *Crr5* in Chinese cabbage

**DOI:** 10.1093/hr/uhae338

**Published:** 2024-12-04

**Authors:** Shuangjuan Yang, Xiangfeng Wang, Zhaojun Wang, Wenjing Zhang, Henan Su, Xiaochun Wei, Yanyan Zhao, Zhiyong Wang, Xiaowei Zhang, Li Guo, Yuxiang Yuan

**Affiliations:** Institute of Vegetables, Henan Academy of Agricultural Sciences, No.116 Huanyuan Road, Jinshui District, Zhengzhou, Henan 450002, China; Shandong Key Laboratory of Precision Molecular Crop Design and Breeding, Peking University Institute of Advanced Agricultural Sciences, Shandong Laboratory of Advanced Agricultural Sciences in Weifang, No.699 Binhu Road, Xiashan District, Weifang, Shandong 261325, China; College of Tobacco Science, Henan Agricultural University, No.218 Ping'an Road, Zhengdong New District, Zhengzhou, Henan 450046, China; Institute of Vegetables, Henan Academy of Agricultural Sciences, No.116 Huanyuan Road, Jinshui District, Zhengzhou, Henan 450002, China; Institute of Vegetables, Henan Academy of Agricultural Sciences, No.116 Huanyuan Road, Jinshui District, Zhengzhou, Henan 450002, China; Institute of Vegetables, Henan Academy of Agricultural Sciences, No.116 Huanyuan Road, Jinshui District, Zhengzhou, Henan 450002, China; Institute of Vegetables, Henan Academy of Agricultural Sciences, No.116 Huanyuan Road, Jinshui District, Zhengzhou, Henan 450002, China; Institute of Vegetables, Henan Academy of Agricultural Sciences, No.116 Huanyuan Road, Jinshui District, Zhengzhou, Henan 450002, China; Institute of Vegetables, Henan Academy of Agricultural Sciences, No.116 Huanyuan Road, Jinshui District, Zhengzhou, Henan 450002, China; Shandong Key Laboratory of Precision Molecular Crop Design and Breeding, Peking University Institute of Advanced Agricultural Sciences, Shandong Laboratory of Advanced Agricultural Sciences in Weifang, No.699 Binhu Road, Xiashan District, Weifang, Shandong 261325, China; Institute of Vegetables, Henan Academy of Agricultural Sciences, No.116 Huanyuan Road, Jinshui District, Zhengzhou, Henan 450002, China

## Abstract

*Brassica rapa* includes a variety of important vegetable and oilseed crops, yet it is significantly challenged by clubroot disease. Notably, the majority of genotypes of *B. rapa* with published genomes exhibit high susceptibility to clubroot disease. The present study presents a high-quality chromosome-level sequence of the genome of the DH40 clubroot-resistant (CR) line, a doubled haploid line derived from the hybrid progeny of a European turnip (ECD01) and two lines of Chinese cabbage. The assembled genome spans 420.92 Mb, with a contig N50 size of 11.97 Mb. Comparative genomics studies revealed that the DH40 line is more closely related to the Chinese cabbage Chiifu than to the turnip ECD04. The DH40 genome provided direct reference and greatly facilitate the map-based cloning of the clubroot resistance gene *Crr5*, encoding a nucleotide-binding leucine-rich repeat (NLR) protein. Further functional analysis demonstrated that *Crr5* confers clubroot resistance in both Chinese cabbage and transgenic *Arabidopsis*. It responds to inoculation with *Plasmodiophora brassicae* and is expressed in both roots and leaves. Subcellular localization shows that Crr5 is present in the nucleus. Notably, the Toll/interleukin-1 receptor (TIR) domain of Crr5 can autoactivate and trigger cell death. In addition, we developed two Crr5-specific Kompetitive allele-specific PCR (KASP) markers and showcased their successful application in breeding CR Chinese cabbage through marker-assisted selection. Overall, our research offers valuable resources for genetic and genomic studies in *B. rapa* and deepens our understanding of the molecular mechanisms underlying clubroot resistance against *P. brassicae*.

## Introduction

The *Brassica* genus includes a variety of significant vegetables and oilseed plants, including Chinese cabbages, turnips, cabbages, cauliflowers, and oilseed rape. Six *Brassica* species, namely, *Brassica rapa* (AA), *Brassica nigra* (BB), *Brassica oleracea* (CC), *Brassica juncea* (AABB), *Brassica napus* (AACC), and *Brassica carinata* (BBCC), form the well-established U’s triangle that explains the genomic associations among three diploid and three allopolyploid *Brassica* species (Nagaharu, 1935) [1]. *Brassica rapa* is a key agricultural crop that consists of several subspecies with diverse morphotypes, including heading (ssp. *pekinensis*) and nonheading (ssp. *chinensis*) Chinese cabbage, enlarged turnip (ssp. *rapifera*), and the seed oil crop yellow sarson (ssp. *trilocularis*) [2, 3]. The heading Chinese cabbage is further categorized into overlapped, closed, spiraled, and outward-curving heading types [4, 5]. The nonheading Chinese cabbage comprises pak-choi (var. *communis* Tesn et Lee), Caitai (var. *tsai-tai* Hort.), Taicai (var. *tai-tsai* Hort.), Tacai (var. *rosularis* Tsen et Lee), and Fenniecai (var. *multiceps* Hort.) [6]. The high variability between two morphotypes of the same species of *Brassica* highlights the significance of determining several reference assemblies for a particular species [7]. Recently, three Telomere-to-Telomere (T2T) genome assemblies of *B. rapa*, namely one Chinese cabbage ‘Chiifu-401-42’, and two nonheading Chinese cabbages ‘AiJiaoBai’ and ‘HongShanCaiTai’, were published [8, 9]. In addition, chromosome-level genome sequences of Chinese cabbage ‘A03’ [5], four nonheading Chinese cabbage ‘NHCC001’ [6], ‘ZYCX’ [10], ‘PC-fu’ ,[3], and ‘XiangQingcai’ [11], one turnip ‘ECD04’ [12], and one yellow sarson ‘Z1’ [7], have been recently reported. The genomes of these species serve an abundant data source for the genetic and genomic analyses of *Brassica* species. However, only two Chinese cabbage genotypes, having high susceptibility to clubroot disease, have been *de novo* assembled to date.

The obligate biotrophic parasite, *Plasmodiophora brassicae* Woronin, is the causative agent of clubroot disease, which poses as a serious threat and specifically affects the global production of crops in the Brassicaceae family [13]. Clubroot disease generally reduces the yield of *Brassica* crops by 10%–15%, and can result in losses >40% or complete crop failure during severe outbreaks [12, 14]. *Plasmodiophora brassicae* is a biotrophic pathogen that is capable of surviving for nearly 20 years in soils, which poses as a serious challenge to the control of clubroot disease [15]. Genetic resistance is regarded as the most effective and economic strategy for controlling clubroot. Some qualitative and quantitative clubroot-resistant (CR) loci have been discovered in *B. rapa*, *B. oleracea*, *B. napus*, and *B. nigra* [16]. Over 20 CR loci have been identified in *B. rapa*, which are distributed on chromosomes A01, A02, A03, A05, A06, A07, and A08 [17]. Particularly, CR loci on A03 and A08 are thoroughly investigated, including *CRa*, *CRb*, *CRd*, *CRk*, *CRq*, *Crr3*, *Rcr1*, *Rcr2*, *Rcr4*, and *Rcr5* on A03, and *Crr1a*, *Crr1b*, *CRs*, *Rcr3*, and *Rcr9* on A08 [16, 17]. However, most of these CR loci were reported at the preliminary mapping level, and only two CR loci, *CRa* and *Crr1a*, were successfully isolated and functionally validated [18, 19]. In addition, breakdown of resistance on some of CR cultivars have all been reported in *B. rapa*, *B. oleracea*, and *B. napus* [20]. Thus, the identification of novel CR genes or alleles related to resistance to various pathotypes is crucial for combining multiple CR genes in a single variety for controlling the spread of clubroot disease.

In this study, using a combination of Pacific Biosciences HiFi (PacBio HiFi), next generation sequencing (NGS), and high-throughput chromosome conformation capture and sequencing (Hi-C) technologies, we *de novo* assembled a chromosome-level reference genome of Chinese cabbage doubled haploid line DH40, which was resistant to clubroot disease. Comparison of the genome of DH40 and the published genomes of the Chiifu and ECD04 varieties led to the identification of several single-nucleotide polymorphisms (SNPs), small insertions/deletions (InDels), and structure variations (SVs). Based on the assembly of DH40, bulked segregant analysis (BSA) with NGS combined with map-based cloning led to the identification of the *DH40A08G013380* (*Crr5*) gene, which encodes a nucleotide-binding leucine-rich repeat (NLR) protein, and is the causal gene that controls the clubroot resistance trait in DH40. Furthermore, we investigated the expression pattern, subcellular localization, and function of *Crr5*. In addition, we developed gene-specific markers for *Crr5* and applied these markers in clubroot resistance breeding. The results obtained herein will serve as key genomic resources for future genomics-based analysis of Brassicaceae and shed light on the molecular mechanism underlying *Crr5*-mediated resistance and its application on breeding in *Brassica* species.

## Results

### *De novo* assembly and annotation of the DH40 genome

**Figure 1:**
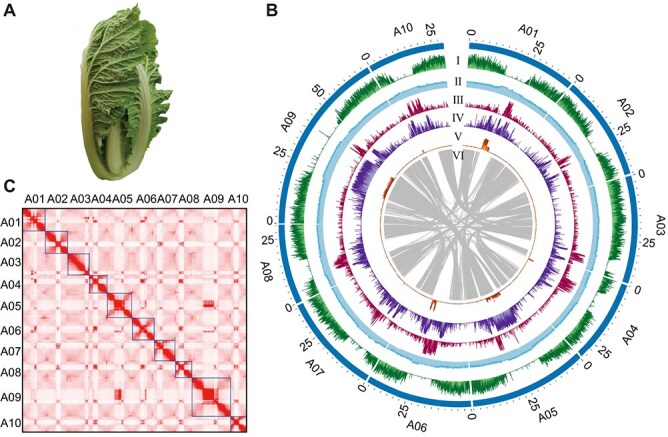
Overview of the DH40 features. (A) A photograph of DH40 plant. (B) Circos plot shows gene density (I), GC content (II), *Copia* element density (III), *Gypsy* element density (IV), noncoding RNA density (V), and syntenic blocks (VI) in DH40 genome. (C) Hi-C interaction among 10 chromosomes.

The CR doubled haploid DH40 line (Fig. 1a) was selected herein for genome sequencing and assembly. Genomic survey using *k*-mer frequency analysis of 32.46 Gb clean short reads revealed that the size of the DH40 genome was ~465.71 Mb, having a ~0.215% heterozygosity and a repeat content of 54.25% (Table S1). The 19.02 Gb (~40×) PacBio HiFi reads (Table S2) obtained were employed for the initial assembly of the DH40 genome using hifiasm [21], Flye [22] and NextDenovo [23]. The two assemblies from Flye and NextDenovo were used to correct errors in hifiasm assembly and subsequently polished by HiFi and NGS reads, obtaining a draft genome of 420.92 Mb in size, representing 90.38% of the estimated genome. The assembled genome contained 124 contigs of which contig N50 had a size of 11.97 Mb (Table 1). The 116.02 Gb Hi-C data was subsequently used to scaffold the initial assembly into 10 chromosomes having a size totaling 408.75 Mb (Tables 1; Table S3), with the remaining 12.21 Mb as unplaced contigs. The intrachromosomal interactions in the Hi-C interaction matrices exhibited a distinct antidiagonal pattern and showed no sign of large misplaced/oriented contigs (Fig. 1c). To evaluate the quality of this genome assembly, Benchmarking Universal Single-Copy Orthologs (BUSCOs) were assessed showing 99.32% complete BUSCOs, including 1376 single-copy orthologs and 238 duplicated orthologs (Table S4). Additionally, the quality value (QV) was 51.03 suggesting a high completeness. In addition, 97.3% of the NGS reads and 99.87% of the HiFi reads could be mapped to the DH40 genome. Collectively, the findings substantiated that the DH40 genome exhibited both high continuity and comprehensiveness.

**Table 1:** Statistics and results of annotation of the assembled DH40 genome.

Assembly and annotation feature	Statistics
Assembled genome size (Mb)	420.92
Contig number	124
Contig N50 (Mb)	11.97
Contig max (Mb)	35.82
Scaffold number	32
Scaffold N50 (Mb)	40.76
Anchored to chromosome (Mb)	408.75
GC content (%)	37.44
Total repetitive sequences (%)	53.07
Annotated protein-coding genes	45 863
Average length of coding sequence (bp)	1112.4
Average number of exons per gene	5.18
Completeness (%, BUSCO)	99.32

Genome annotation using *ab initio*, homology-based, and transcriptome-based approaches led to the prediction of 45 863 protein-coding genes (Fig. 1b; Table 1), among which, 44 751 (97.57%) were annotated in a minimum of one protein function database (Table S5). The vast majority of genes (99.54%) were anchored on chromosomes, leaving only 0.46% on unplaced scaffolds. A total of 223.39 Mb (53.07%) repetitive elements (Fig. 1b) were annotated in DH40 genome using *de novo*- and homology-based strategies (Table 1), which was comparable to the near-complete genome of Chiifu v4.0 (53.78%), but higher than those less complete genomes Chiifu v3.0 (45.84%) [24], Chinese cabbage ‘A03’ (50.99%) [5], Pak choi ‘NHCC001’ (46.15%) [6], and turnip ECD04 (42.71%) [12]. Long terminal repeat (LTR) retrotransposons were the most predominant repetitive elements, constituting 19.64% of the genome, followed by satellites (10.86%), DNA transposons (5.01%), and LINEs (2.40%) (Table S6). In addition, the results of genome annotation revealed that 0.99% (4.17 Mb) comprised noncoding RNAs, including 421 miRNAs, 1148 tRNAs, 6627 rRNAs, and 1338 snoRNAs (Table S7).

### Comparison of DH40 with Chiifu and ECD04 genome

**Figure 2:**
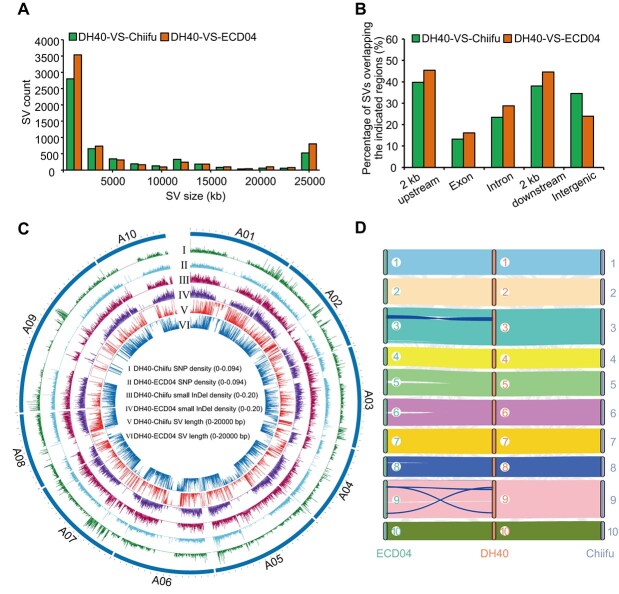
Comparison of the DH40 genome with Chiifu V 4.0 and ECD04 genome. (A) Distribution of SV size. (B) The proportion of SVs that mapped to different genomic regions. (C) Circos plot shows SNPs, small InDels, and SVs among DH40 with Chiifu V4.0 and ECD04 genome. (D) Syntenic comparison of DH40 with Chiifu V 4.0 and ECD04 genome. Syntenic blocks on the same chromosomes of two genomes were linked with colored lines, and that on the different chromosomes were linked with gray lines.

Considering DH40 as a progeny of European turnip and two Chinese cabbage lines, we compared the DH40 genome to that of Chiifu (Chinese cabbage) and ECD04 (European turnip). Using DH40 as reference, we aligned the genomes of Chiifu V4.0 and ECD04 to identify the genetic variations, namely, SNPs, small InDels (<50 bp), and SVs (≥50 bp), which led to the identification of 1 290 313 SNPs and 454 111 small InDels between DH40 and Chiifu V4.0, with an average of 3.15 SNPs and 1.11 small InDels per kb (Fig. 2c), respectively. Among which, 205 089 (15.89%) SNPs were located in the exonic region, 75 468 (5.85%) were nonsynonymous SNPs (Tables S8 and S9), and 12 036 (2.65%) small InDels led to the generation of nonsynonymous, splicing sites, and frameshifts (Tables S10 and S11). As for the comparison between DH40 and ECD04, 1 438 758 SNPs (Table S12) and 391 045 small InDels (Table S13) were detected in the aligned syntenic blocks, and the average numbers of SNPs and InDels were 3.5 and 0.95 per kb, respectively (Fig. 2c). Among which, 320 980 (22.31%) SNPs were mapped to the coding region (Table S12), 115 701 (8.42%) were nonsynonymous SNPs (Table S14), and 10 120 (2.58%) small Indels caused changes of nonsynonmous, frameshifts, or splicing sites (Table S15).

A total of 5402 SVs (50 bp–8.38 Mb in size) were detected between the genomes of DH40 and Chiifu V4.0 (Fig. 2a), including 2685 insertions, 2610 deletions, 64 inversions, and 43 translocations (Table S16), which together covered 45.02% (184.02 Mb) of the total genome. By contrast, 6370 SVs ranging from 50 bp to 3.66 Mb were identified between DH40 and ECD04, including 3121 insertions, 3117 deletions, 91 inversions, and 41 translocations (Table S17), which together covered 56.61% (231.41 Mb) of the total genome. SVs can affect the gene-coding regions to disrupt their functions. As shown in Fig. 2b, 13.38% (723 out of 5402) and 16.31% (1039 out of 6370) of the annotated SVs were overlapped with the exon sequences, which were markedly less compared to the respective abundances in the intergenic regions, and the findings agreed with those of previous studies [25–27]. Clearly, the number of variants between DH40 and Chiifu V4.0 (1 290 313 SNPs, 454 111 SNPs, and 5402 SVs) was overall lower than that between DH40 and ECD04 (1 438 758 SNPs, 391 045 small InDels, and 6370 SVs). Moreover, the length of SVs between DH40 and Chiifu V4.0 (184.02 Mb) was much shorter than between DH40 and ECD04 (231.41 Mb), which suggested that the DH40 was more closely related to Chiifu than ECD04 in genetic background, and which was coincided with the fact that DH40 and Chiifu were both Chinese cabbage morphotypes, while ECD04 was turnip morphotype.

Despite the observed genomic variations, synteny analysis indicated that the genome of DH40 shared strong genetic collinearity with the two other *B. rapa* genomes, especially Chiifu (Fig. 2d). The synteny also confirmed that the DH40 was closer to Chiifu than to ECD04. There were significant regions of chromosomal rearrangements, deletions, and insertions between DH40 and turnip ECD04. The variations delineated the genomic regions into 75 syntenic blocks based on relative gene positions and sequence similarity (Table S18). Little chromosomal rearrangements were detected between DH40 and Chiifu, which shared 55 collinear blocks that were generally larger and more continuous than those between DH40 and ECD04 (Fig. 2d; Table S18). For example, there were only two and four syntenic blocks between DH40 and Chiifu V4.0 on chromosome A03 and A09, respectively, whereas there were 9 and 12 syntenic blocks between DH40 and ECD04 (Table S18; Fig. 2d). The impact of these variations was also reflected in the number of syntenic gene pairs between the genomes, with DH40 sharing 80.91% (35 111 pairs) with Chiifu, while sharing 76.52% (33 206 pairs) with ECD04 (Table S18).

### Rapid mapping of the *Crr5* CR gene in DH40 by BSA

**Figure 3:**
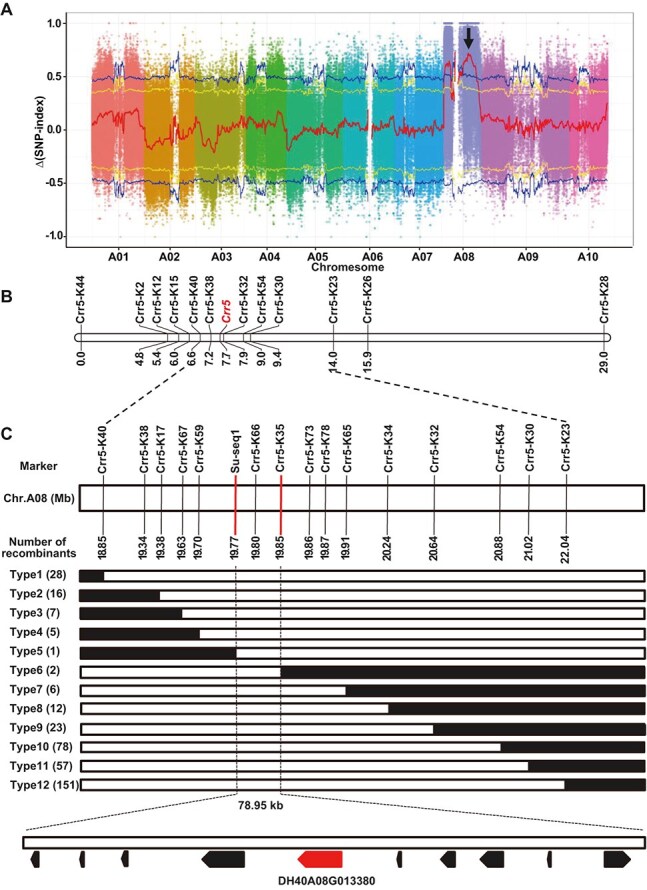
Map-based cloning of *Crr5* gene in Chinese cabbage. (A) BSA-seq analysis for *Crr5*. Two candidate regions were identified on chromosome A08 with peak at 19.55 Mb (arrow indicated). (B) Initial mapping of *Crr5*. Genetic map unit, cM. (C) Fine mapping of *Crr5*. Genetic structure of the recombinant types; the homozygous susceptible genotypes and heterozygous alleles are depicted in white and black, respectively. The number of each recombinant type is indicated in brackets.

The genetic basis of clubroot resistance in DH40 was evaluated by constructing a segregating population by crossing the clubroot-susceptible DH199 line with DH40. The F_1_ plants all displayed a resistance phenotype. The F_2_ progeny comprised resistant and susceptible plants in a 3:1 ratio (Table S19), suggesting a single dominant gene controls clubroot resistance in DH40. The CR gene was subsequently denoted as *Crr5*. To rapidly map the *Crr5* gene, a BSA-seq analysis was conducted using the resistant (R-pool) and susceptible (S-pool) F_2_ individuals. A total of 114 308 002 and 89 049 098 clean reads (Table S20) were obtained from the R- and S-pools, respectively. Using the DH40 genome as reference, 1 755 293 SNPs and 220 352 InDels were identified between the R- and S-pools. Sliding window analysis with the △(SNP-index) revealed two candidate regions on chromosome A08 at the 99% confidence level (Table S21), with a peak at 19.55 Mb, at which the SNP indexes for the S- and R-pools were 0.94 and 0.22, respectively (Fig. 3a). Using BSA-seq analysis, we developed 18 Kompetitive allele-specific PCR (KASP) markers and genotyped on 94 F_2_ individuals for linkage analysis (Tables S22 and S23). The results revealed that *Crr5* gene cosegregated with the markers Crr5-K38 and Crr5-K32 (Fig. 3b; Table S23). The *Crr5* locus was located 0.5 and 0.2 cM from Crr5-K38 and Crr5-K32, respectively.

**Figure 4:**
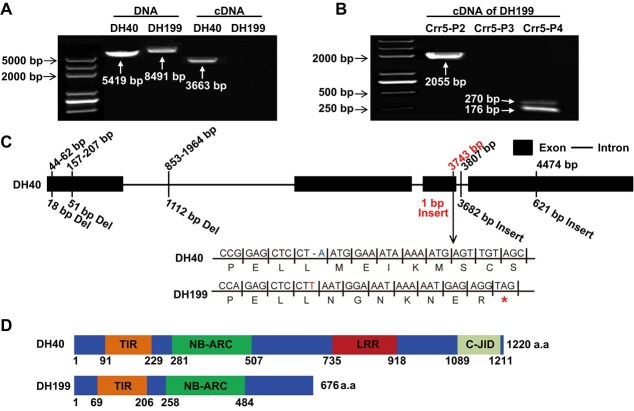
Candidate gene analysis of *Crr5*. (A) Products of amplification using the full-length primer with DNA and cDNA from DH40 and DH199. The full-length CDS of the susceptible DH199 line was undetectable. (B) Amplification products of each fragmental primer using cDNA from DH199. Primer Crr5-P2 spanned from exon 1 to exon 3 and this region was transcribed. Primer Crr5-P3 spanned from the end of exon 3 to exon 4 and this region was not transcribed. Primer Crr5-P4 spanned the intron 2, alternative splicing happened in this region. Primer sequences were listed in Table S25. (C) The *Crr5* gene includes four exons and three introns. The positions upon the gene structure diagram were based on the DNA sequence of *Crr5* in DH40. Insertions and deletions of DH199 DNA sequence relative to DH40 were depicted at the bottom of the gene structure diagram. In the susceptible DH199, the insertion of a single nucleotide (T) led to the generation of a termination codon prematurely (marked with asterisks) caused by a frameshift mutation. (D) Structures of the Crr5 proteins of DH40 and DH199. The SNP insertion in DH199 eliminated the LRR and C-JID domains at the C-terminus.

The target region was further narrowed down by screening 1412 susceptible individuals from the total 5700 F_2_ plants. Using markers Crr5-K40 and Crr5-K23, 28 and 151 recombinants (Fig. 3c) were detected, respectively. Furthermore, 10 new markers were developed to detect the recombination events (Table S22). The *Crr5* was flanked by Crr5-K40, Crr5-K38, Crr5-K17, Crr5-K67, Crr5-K59, and Su1-seq1 on one side and Crr5-K35, Crr5-K73, Crr5-K78, Crr5-K65, Crr5-K34, Crr5-K32, Crr5-K54, Crr5-K30, and Crr5-K23 on the other side. The *Crr5* gene was delimited to a 78.95-kb region (A08: 19774426-19 853 376 bp) flanked by the Su1-seq1 and Crr5-K35 markers (Fig. 3c). The marker Crr5-K66 cosegregated with *Crr5.* DH40 genome annotation showed that 10 genes were located among the 78.95-kb candidate region of *Crr5* (Table 2), among which, the *DH40A08G013380* gene was selected the most probable candidate as it encoded a TIR-NBS-LRR protein (Table 2). The genomic and coding sequences (CDS) of *DH40A08G013380* were amplified and sequenced from the parental lines with the Crr5-fulF and Crr5-fulR primer pairs (Table S24). The results confirmed that the *DH40A08G013380* gene of the resistant DH40 parent was 5419 bp (3663 bp CDS), consisting of four exons and three introns (Fig. 4A and  4B). By contrast, in susceptible parent DH199, the *DH40A08G013380* gene was 8491 bp in length, and the full-length CDS could not be amplified (Fig. 4B). Sequence alignment analysis led to the identification of 169 SNPs and 17 InDels between the genomic sequence of DH40 and DH199 (Figs. S3–S9; Table S25). Among these variations, an insertion of thymine at 3743 bp (T3743) caused a frameshift mutation that led to the generation of a termination codon prematurely at residue 676 (Fig. 4C and  4D). Conserved domain analysis using the EBI webserver (https://www.ebi.ac.uk/interpro) showed that the *DH40A08G013380* gene of DH40 contained a Toll/interleukin-1 receptor (TIR) domain (91–229 a.a.), an NB-ARC domain (281–507 a.a.), LRR domain (735–918 a.a.), and C-JID domain (1089–1211 a.a.) (Fig. 4d). The T3743 in DH199 led to the elimination of the two conserved domains at the C-terminal, leading to the loss of function of *Crr5* (Fig. 4d). Another reason underlying the loss of function of *Crr5* was the retention of the second intron at the transcriptional level (Fig. 4b), which led to the generation of a stop codon prematurely at the translational level.

**Table 2:** Genes annotated in the candidate interval of the *Crr5* locus.

Gene	Location on A08	*Arabidopsis* homolog	Annotation
*DH40A08G013340*	19 775 068–19 775 889		Uncharacterized protein
*DH40A08G013350*	19 780 887–19 781 200		Uncharacterized protein
*DH40A08G013360*	19 790 936–19 791 600	*AT3G28540*	Belongs to the AAA ATPase family
*DH40A08G013370*	19 798 974–19 804 155	*AT4G21910*	MATE efflux family protein
*DH40A08G013380*	19 811 269–19 816 687	*AT5G11250*	Disease resistance protein; atypical TIR-NBS-LRR
*DH40A08G013390*	19 823 497–19 823 709		Uncharacterized protein
*DH40A08G013400*	19 829 282–19 831 031	*AT4G22080*	Pectate lyase 16
*DH40A08G013410*	19 833 354–19 836 241	*AT4G22120*	Calcium-permeable stretch-activated cation channel
*DH40A08G013420*	19 842 249–19 842 569	*AT5G16000*	NSP-interacting kinase (NIK1), receptor-like kinase, mediates defense response against geminivirus
*DH40A08G013430*	19 849 642–19 852 890	*AT4G22130*	STRUBBELIG-receptor family 8

### Functional characterization of *Crr5*

**Figure 5:**
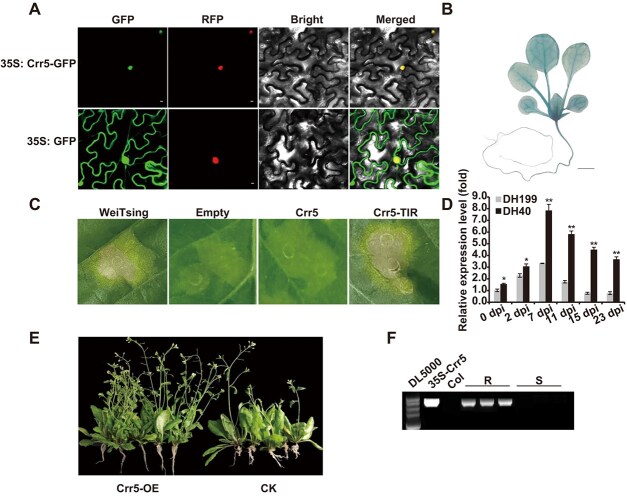
Functional analysis of *Crr5*. (A) Subcellular localization analysis of *Crr5*. Nucleus marker protein pNLS-RFP was employed as the positive control. (B) Spatial expression of *Crr5* indicated by GUS staining in 14-day-old Crr5Pro::GUS seedlings. (C) Transient expression of the TIR domain of Crr5 could induce cell death in tobacco leaves, while the full length Crr5 could not elicit cell death response. The *WeiTsing* gene and the empty construct served as the positive and negative control, respectively. (D) Relative expression level of *Crr5* in resistant DH40 and in susceptible DH199 at different days postinoculation of *PbXY-2* isolates. *BrGAPDH* served as the internal control. The error bars denote SE (*n* = 3). * *P* < 0.05, ** *P* < 0.01. (E) Overexpression of *Crr5* in Arabidopsis Col-0 showed the CR phenotype. (F) Results of genotyping of the resistant and susceptible T_2_ progeny. R and S denote resistant and susceptible lines, respectively. The 35S-Crr5 plasmid and the wild-type Col-0 served as the positive and negative controls, respectively.

In order to understand the functions of *Crr5*, its subcellular localization was initially predicted computationally using the web-based WoLF-PSORT tool (https://wolfpsort.hgc.jp). The Crr5 was predicted to localize in the nucleus (scores of nucleus, cytoplasm, and chloroplast were 7, 3, and 1, respectively). Then we conducted experimental validation by transiently expressing a fused Crr5-green fluorescent protein (GFP) construct, which revealed that Crr5 distinctly colocalized in the cellular nucleus as indicated by pNLS-RFP, in contrast to the GFP control signals that were prevalent throughout the foliar cells of tobacco (Fig. 5a). The spatial expression of *Crr5* was further explored by introducing a GUS reporter gene driven by the *Crr5* promoter into *Arabidopsis*. Histochemical staining of T_2_ transgenic plants revealed GUS expression in both leaves and roots, notably at the root–stem junction (Fig. 5b). Furthermore, we assessed the expression pattern of *Crr5* in both the resistant DH40 line and the susceptible DH199 line during infection by PbXY-2 isolate, employing the primer pairs Crr5-qPCR-F and Crr5-qPCR-R (Table S24). *Crr5* expression gradually increased over the course of the infection in both DH40 and DH199, peaking at 7 dpi (days postinoculation), and gradually decreased thereafter (Fig. 5d). Notably, *Crr5* expression was significantly upregulated in the resistant DH40 line throughout all infection stages, especially at 7 dpi (Fig. 5d), indicating a positive correlation between *Crr5* expression and clubroot resistance in Chinese cabbage.

To deepen our understanding of *Crr5*’s function, we transiently overexpressed *Crr5* in tobacco (*Nicotiana tabacum*). The overexpression of the full-length *Crr5* in tobacco leaves failed to trigger cell death at the injection area. However, the N-terminal region of *Crr5*, including the TIR domain (N-TIR), triggered a noticeable cell death response following expression (Fig. 5c). To further examine the function of *Crr5*, we isolated the full-length CDS of *Crr5* from resistant line DH40 and integrated it into a binary vector regulated by the constitutive CaMV 35S promoter with stable transformation of *Arabidopsis thaliana*. The resistance of the T_1_ seeds of the 16 independent transgenic plants (T_0_) to PbXY-2 isolate were evaluated. Three of the T_0_ lines produced resistant T_1_ plants (Fig. 5e). The resistant T_1_ plants were selected for propagation, and the existence and nonexistence of exogenous *Crr5* were evaluated in the obtained T_2_ plants. The findings revealed that the resistant T_2_ plants harbored the *Crr5* gene, while the susceptible T_2_ plants did not possess the *Crr5* gene (Fig. 5f), suggesting that *Crr5* functions as a CR gene.

### Development of *Crr5*-specific markers and its application in breeding

We then sought to apply the *Crr5* gene in marker-assisted breeding of Chinese cabbage for clubroot resistance by developing and implementing a *Crr5*-specific marker. To do so, we firstly designed the KASP maker Crr5-funK1 (Table S22) based on the aforementioned T3743 insertion. We validated the Crr5-funK1 on the (DH40 × DH199)-F_2_ population, which contained 39 resistant plants and 47 susceptible plants, and on a natural population, which contained DH40 and 47 susceptible DH or inbred lines (Table S26). We observed that Crr5-funK1 cosegregated with the CR phenotype in the (DH40 × DH199)-F_2_ population (Fig. S10a). By contrast, in the natural population, only 10 susceptible lines showed the same genotype with DH199 and other 37 susceptible lines exhibited the same genotype with DH40 (Fig. S10b; Table S26), suggesting that the T 3743 insertion in DH199 was not a common feature in the susceptible lines and thus Crr5-funK1 could not be used as a gene-specific marker for selecting resistant allele.

**Figure 6:**
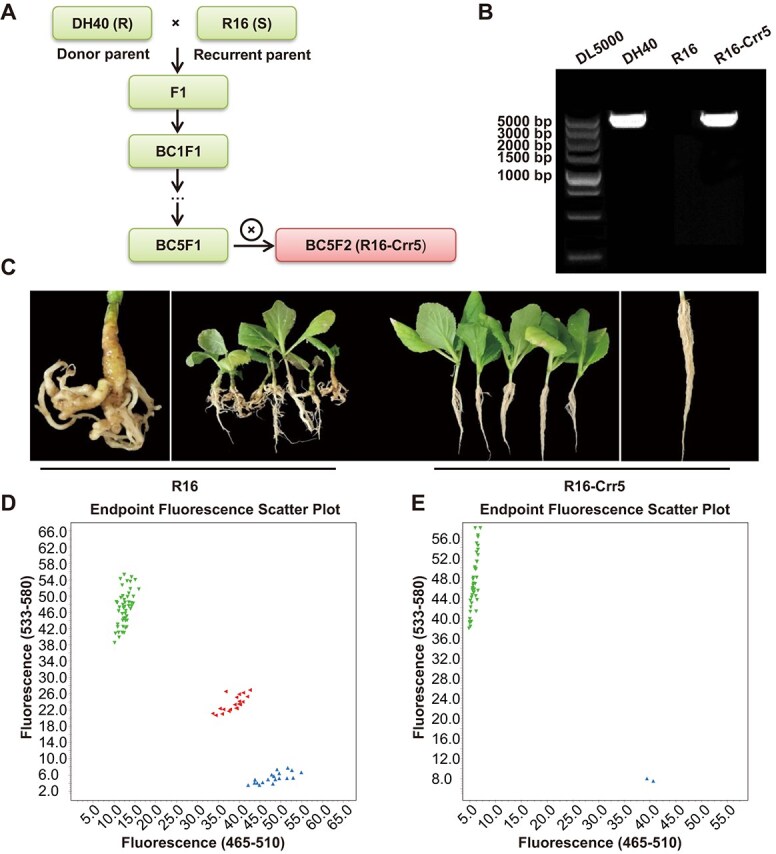
Application of *Crr5*-specific marker in molecular-assisted breeding. (A) Development of the resistant line R16-Crr5 using the Crr5-specific marker-assisted selection. DH40 was used as the donor parent and susceptible R16 was used as the recurrent parent. (B) Validation of the presence of *Crr5* in the R16-Crr5 genome by PCR. (C) Clubroot phenotype of R16 and R16-Crr5 after inoculation of *PbXY-2* isolate. Gall formation was examined and photographed at 35 days after inoculation. (D, E) Validation of the Crr5-specific marker in (DH40 × DH199)-F_2_ population (D) and in a natural population (E).

To develop a common sequence variation between susceptible and resistant materials, we aligned the resequencing data of four susceptible lines, namely, YW81, R16, Y640–288, and Y641–87, to the DH40 genome and called SNPs between these five materials. We selected the SNPs showing the same genotype in the four susceptible lines but with polymorphism between the resistant line DH40. The results showed that there were 630 SNPs between these five materials among the *Crr5* region (19 811 269–19 816 687 bp), but only 15 SNPs satisfied the above criteria (Table S27). According to the KASP marker development procedure [4], 2 of the 15 SNPs were converted to KASP markers, Crr5-funK3 and Crr5-funK4 (Table S22), cosegregated with the CR phenotype not only in the (DH40 × DH199)-F_2_ population (Fig. 6d) but also in the natural population (Fig. 6e), indicating Crr5-funK3 and Crr5-funK4 could be used as the Crr5-specific markers. Using marker Crr5-funK3 as foreground selection, and using DH40 as donor parent, we introduced the *Crr5* gene into the susceptible line R16 and developed a near-isogenic line R16-Crr5 through five generations of backcrossing (Fig. 6a), whose proportion of recurrent parent genome (PRPG) was high to 96.7%. Using R16 as the susceptible control, its near-isogenic line R16-Crr5 owned the *Crr5* full-length DNA fragment (Fig. 6b) and showed complete resistance to the *PbXY-2* isolate (Fig. 6c).

## Discussion

Genome sequencing has played a crucial role in advancing various facets of basic biology and has various applications in medicine and agriculture [28]. In the past few years, single-molecule DNA sequencing technologies such as PacBio SMRT and Oxford Nanopore sequencing have substantially improved genome assembly quality [28–30]. Nevertheless, these methods, which depend on single-molecule detection, typically have lower read accuracy (75%–90%) [31–33]. Although read-to-read error correction can improve consensus accuracy, it is computationally demanding and some errors may still occur from incorrect read mapping during the correction process [32]. The recently updated HiFi sequence reads from PacBio’s CCS mode achieve a balance between read lengths and quality of the bases [32, 34]. This allows the generation of more complete, contiguous, and accurate chromosome-level genome assemblies and T2T genomes [35, 36]. *Brassica rapa* is an agronomically important species. To date, three T2T *B. rapa* genomes have been reported [8, 9]; the two nonheading Chinese cabbage ‘AiJiaoBai’ and ‘HongShanCaiTai’, which used the HiFi technology, achieved gap-free levels (Table S29). Further, seven other chromosome-level *B. rapa* genomes have also been published [8, 11], and only one genome of ‘XiangQingcai’ was conducted using the PacBio HiFi sequencing (Table S28). In this study, using PacBio HiFi and Hi-C technologies, a high-quality genome of 420.92 Mb having a contig N50 of 11.97 Mb was generated for a CR Chinese cabbage line DH40, which was more continuous than most of the published genomes (Table S29), including the Chinese cabbage ‘A03’ [5], three nonheading Chinese cabbage ‘NHCC001’ [6], ‘ZYCX’ [10], ‘PC-fu’ [3], the turnip ‘ECD04’ [12], and the yellow sarson ‘Z1’ [7]. In addition, the BUSCO value was also higher than those above six *B. rapa* genomes (Table S28), suggesting that the DH40 genome we assembled in this study had high completeness, contiguity, and accuracy. Interestingly, synteny analysis supported that the DH40 was much closer to Chinese cabbage Chiifu than the turnip ECD04, although DH40 derived from the hybrid progeny of a European turnip ECD01 and two Chinese cabbage lines. Genome comparison identified overall more variants between DH40 and ECD04 than between DH40 and Chiifu. Particularly, large-scale genome rearrangements such as a 3.5-Mb inversion between ECD04 and Chiifu [12] were detected between DH40 and ECD04 (Fig. 2d; Table S17), but not between DH40 and Chiifu (Fig. 2d; Table S16). In this pan-genome era, the availability of this high-quality genome of DH40 will advance genetic breeding and enhance evolutionary and comparative studies in *B. rapa*.

The assembled DH40 genome greatly facilitates our discovery of the clubroot resistance gene *Crr5*. When conducting the BSA-seq analysis based on △(SNP-index) method [37], we identified the peak △(SNP-index) at 19.55 Mb on A08 chromosome when using the DH40 as reference, which was quite close to the physical position (19 811 269–19 816 687 bp) of *Crr5* (*DH40A08G013380*) (Fig. 3; Table S21). However, when using Chiifu V4.0 as the reference, we detected two regions on A08 with peak at 15.75 Mb (Fig. S11; Table S29), which was distant from the physical position (20 198 458−20 207 103 bp) of *Crr5* susceptible allele on Chiifu V4.0. This demonstrated the necessity and value of generating a DH40 genome assembly for map-based cloning of resistance gene *Crr5*. For another, when constructing the initial mapping of *Crr5* using linkage analysis, we observed that seven markers (Crr5-K50, Crr5-K2, Bra045–3, Bra049–1, Crr5-KM7, Crr5-KMM10, and Crr5-KM13) spanning from 4.95 to 17.00 Mb displayed identical genotypes among all the 94 individuals (Table S23), suggesting recombination suppression among this region. Large inversions are known to seriously suppress recombination in cabbage [38], tomato [39], and other crops [40, 41]. By examining SVs between DH40 and the other two genomes, we found a large inversion from 7.42 to 12.66 Mb present between DH40 and Chiifu V4.0 but not in DH40 and ECD04 comparison (Fig. S12; Tables S16 and S17). This inversion event might be responsible for the recombination suppression of the above seven markers, which might also affect the efficiency of BSA-seq analysis if using Chiifu V4.0 as a reference.

The innate immunity system of plants consists of pathogen-associated molecular pattern (PAMP)-triggered immunity (PTI) and effector-triggered immunity (ETI) [42]. In ETI, the effectors are identified by NLR proteins in plant cells, which subsequently triggers a potent immune response that is related to programmed cell death, referred to as hypersensitive response (HR), which limits the proliferation of the pathogens [12, 43]. One of the most studied NLR resistance genes in *Arabidopsis* is *RPP1*, which encodes a TIR-NBS-LRR protein. The TNL receptor, RPP1, confers strain-selective immune resistance via recognition of the *Hyaloperonospora arabidopsidis* (*Hpa*) effector, ATR1 [44, 45]. The TIR domain of RPP1 induces HR-related cell death, while the LRR and C-JID domains at the C-terminal of RPP1 interact directly with *Hpa* effector ATR1, which leads to the assembly of the RPP1 tetramer that is necessary for the death of the host cell [44, 46]. In this study, we isolated the CR gene *Crr5* in DH40, which encodes a TIR-NBS-LRR protein. The *Crr5* gene was same with the gene *Crr1a^Kinami90_a^* [47], which was allelic to the already cloned *Crr1a* [19] (Figs. S13 and S14). Additionally, the predicted Crr5 protein was moderately similar to the RPP1 protein of *Arabidopsis* (57.11% identity). *Crr5* is a dominant resistance gene in Chinese cabbage and in overexpressed *Arabidopsis*, but *Crr1a* is an incomplete dominant gene [19]. Protein sequence alignment of Crr5 and Crr1a revealed that there was a high degree of conservation between the TIR and NBS domains, but there were many more variations in the C-terminal LRR and C-JID domains (Fig. S14), which may affect the interaction between *Pb* effectors and the LRR and C-JID domains, and might account for the different levels of resistance conferred by *Crr5* and *Crr1a*. Further investigation is needed to determine the resistance spectrum given by *Crr5* and *Crr1a* against different *Pb* isolates in terms of recognition specificities.

Cell death assay showed that the N-TIR domain of Crr5 rather than the full-length Crr5 elicited HR on tobacco leaves (Fig. 5c), a phenotype reminiscent of *RPP1* gene in *Arabidopsis* [43, 46]. We speculated that Crr5 may act like RPP1, maintaining an inactive state and could not induce HR when *Pb* effectors were absent due to the intramolecular interactions among the N-TIR, NBS, and LRR domains, whereas the N-TIR alone without the inhibition by NBS and LRR domains could be autoactivated and can form homodimers to elicit cell death [43]. GUS staining assay revealed the *Crr5* gene expressed both in roots and leaves, which was aligned with that in *Crr1a* [19]. Expression pattern in different stage after inoculation of *PbXY-2* showed that the *Crr5* gene responded with the inoculation and reached its highest level at 7 dpi, a time from which the secondary infection phase (cortical infection) of *Pb* begins [48]. Both subcellular localization experiment and bioinformatic prediction revealed the Crr5 was localized to nucleus, similar to another TNL resistance gene *BrRPP1* in *B. rapa* [49], the *GhDSC1* in *Gossypium hirsutum* [50], and the *RPP4* and *RPP8* in *Arabidopsis* [51], but different with clubroot resistance gene *CRa* in plasma membrane and cytoplasm [52], *RPP1-WsA* in endoplasmic reticulum and Golgi apparatus, and *RPP1-WsA* in plasma membrane [51]. The detailed molecular mechanisms underlying the *Crr5*-mediated host resistance and how it interacts with pathogen effectors remains to be investigated.

In conclusion, we *de novo* assembled a CR line DH40 in Chinese cabbage, which is of high quality. The assembly of DH40 provided direct reference and greatly facilitated the map-based cloning of the CR gene, *Crr5*, in DH40. Characterization of Crr5 through subcellular location assay, cell death assay, and expression pattern analysis expanded our insights into the molecular mechanisms by which *Crr5* mediates resistance to *P. brassicae*. Most importantly, we developed two Crr5-specific KASP markers and successfully constructed a resistant line R16-Crr5 through marker-assisted selection and backcrossing. These results serve as a significant resource for genetic and genomic investigations in *B. rapa*, and are expected to facilitate the breeding of CR varieties. The findings will also contribute to research on the molecular mechanism underlying the clubroot resistance of different species of Brassicaceae.

## Materials and methods

### Plant materials and sequencing

A doubled haploid line DH40 was *de novo* assembled in this study. The line DH40 was resistant to clubroot and derived from the hybrid offspring of a European turnip ECD01 and two Chinese cabbage lines, namely, Y510–9 and Y177–12. The pedigree of DH40 was displayed in Fig. S1. The leaves of DH40 were used for the extraction of genomic DNA, which was then fragmented. The libraries for genome sequencing were constructed according to the manufacturer’s technique and sequenced on an MGISEQ-2000 sequencing platform (BGI Shenzhen, Guangdong, China) using a 150-bp PE strategy. These data were used for estimating the genome size and assessing the completeness of the final assembly.

A PacBio library with an insert size of 20 kb was constructed using the Pacific Biosciences SMRTbell Express Template Prep Kit 2.0, following the manufacturer’s protocols. The HiFi reads were then generated using the PacBio Sequel II platform (BGI Shenzhen, Guangdong, China). The Hi-C library was prepared as per the standard protocol. Briefly, the fresh leaves were fixed in formaldehyde and subjected to lysis. Following this, the cross-linked DNA was enzymatically digested with MboI. The resulting sticky ends of the DNA were biotin-tagged, which facilitated their adjacent joining to create a chimeric linkage. Subsequently, these chimeras were physically fragmented to achieve a size range of 300–500 base pairs (bp). An MGISEQ-2000 platform was finally used for library sequencing in the PE150 mode.

### Genome assembly and quality evaluation

After removal of adaptor sequences, the PacBio HiFi reads having an average length of 15.45 Kb, N50 of 15.68 Kb, and a total size of 19.02 Gb were used for genome assembly. Firstly, we generated genome assemblies by Hifiasm (v0.16.1) [21], Flye (v2.9) [22], and NextDenovo (v2.5.0) [23], respectively. Then, we used the Hifiasm assembly as a reference and employed the software Quickmerge (v0.3) [53] to align the assemblies from Flye and NextDenovo to the reference genome sequentially. The assembly generated using Quickmerge was polished with NextPolish (v1.4.0) [54] using NGS and HiFi reads. Finally, a draft assembly of the genome, comprising 124 contigs, was constructed. These contigs served as the foundation for Hi-C scaffolding, During the Hi-C scaffolding process, the clean Hi-C reads were initially aligned to the contigs using Juicer [55]. Subsequently, the contigs underwent correction, clustering, ordering, and orientation through the 3D-DNA pipeline [56]. Juicebox v1.5 [57] was used for visualizing the interaction heat map of the Hi-C contact frequency matrix and for manual correction of some misjoins.

The completeness of the assembly was evaluated with BUSCO v4.0.6 [58], using the embryophyta_odb10 dataset. The QV was determined with Merqury (v1.3), with k = 21 counts [59]. The quality of the genome assembly was evaluated by aligning the NGS reads to the polished assembly with BWA-MEM [60], using default settings. The genome was also evaluated using the corrected PacBio subreads with minimap2 [61].

### Genome annotation

To predict gene models, we adopted a comprehensive pipeline combining *ab initio* prediction, analysis of homologous proteins, and transcriptome-based evidence. The GeneMark-ET model was initially trained using BRAKER2 v2.1.6 [62] for *ab initio* prediction. The SNAP semi-HMM model was subsequently trained with MAKER v3.01.03 [63]. For homologous protein evidence, protein sequences from *A. thaliana* and *B. rapa* were mapped to the DH40 genome using TBLASTN. In terms of transcriptome evidence, we aligned RNA-seq reads to our genome assembly using HISAT2 [64] and conducted reference-based assembly and *de novo* assembly of transcriptomes using Trinity (v2.8.4) [65]. All the above evidence was integrated using MAKER [63] to predict the final gene models.

The functions of the predicted genes were annotated using BLASTp (BLAST2.11.0 +) and the protein sequences were aligned to the Swiss-Prot and NR public databases. The functions were assigned using these results based on the best hits. The protein domains were identified using the eggNOG-mapper database [66] and the KEGG, GO, and Pfam annotations were determined.

### Repeat annotation

The identification of repeat sequences within the DH40 genome was accomplished by homology-based and *de novo* prediction. For the *de novo* prediction, the RepeatModeler software (v1.0.11) [67] was used for constructing a *de novo* repeat library. Concurrently, the homology-based prediction was achieved by searching the Repbase TE library and the TE protein database (https://www.girinst.org/server/RepBase/index.php) against the DH40 genome with the RepeatMasker software (v4.1.2.p1) [68].

The genome-wide noncoding RNAs, including tRNAs, rRNAs, miRNAs, and snRNAs, were identified using Rfam/Infernal (v.1.1.4) [69] (parameters: cmscan–cut_ga–rfam–nohmmonly–fmt 2 –clanin –tblout) and tRNAscan-SE (v. 2.0.9) [70] (parameters: -E -X 20 –f -m -b -j–detail) based on the homology annotation.

### Comparative genome analysis of DH40, Chiifu, and ECD04

Nucmer v4.0.0 [71] was used for aligning the genomes of Chiifu V4.0 and ECD04 to that of DH40 using the parameter set ‘--maxmatch -t 120 -l 100 -c 500’. The raw alignments results were additionally screened using delta-filter with parameter ‘-m -i 90 -l 100’. The obtained filtered delta files were utilized for detecting the SNPs, InDels, and SVs with SyRI (v1.6.3) pipeline, using default parameters [72]. The detected variations from SyRI were classified into three types of variations, namely, SNPs, InDels, and SVs. The SNPs and small InDels were functionally annotated with the ANNOVAR package [73]. SVs were further classified into four types of variations: insertions, deletions, inversions, and translocations, which were performed as previously reported [74].

The analysis of synteny and whole-genome duplication was conducted using JCVI (v1.1.19) [75]. The synteny blocks were identified by executing a comprehensive all-against-all BLAST query and chaining the hits with a cut-off distance of 20 genes. It was additionally ensured that the individual synteny blocks comprised a minimum of five gene pairs. The visualization of these syntenic blocks was accomplished using the MCscan [76].

### Clubroot resistance phenotyping

The XY-2 isolate of *Plasodiophora brassicae* used herein was isolated from diseased Chinese cabbage plants in the field (Xinye, Henan, China). The isolate was reported to belong to pathotype 4 based on Williams classification [77]. The *P. brassicae* suspension was prepared as described previously [16], and the spore concentration was set to 1.0 × 10^8^ spores/ml. A 10-ml aliquot of this resting spore suspension was inoculated around the seeds during sowing, and an additional 10 ml after 10 days of sowing to ensure that the susceptible materials were fully affected and to ensure the accurate phenotyping of clubroot resistance. Furthermore, all the materials were cultivated in a regulated environment at a temperature of 20°C–25°C under a light/dark photoperiod of 16 h/8 h in a light incubator. After 35 days of sowing, infection scoring was conducted using a scale of 0–4 (Fig. S2) as previously described [78]. The plants with infection scores of 0 or 1 were categorized as resistant, and those with scales of 2, 3, or 4 were categorized as susceptible.

### BSA-seq analysis

Two pools of DNA pools were obtained by adding equal quantities of DNA from 30 resistant F_2_ (R-pool) and 30 susceptible F_2_ (S-pool) individuals for BSA-seq. Sequencing was performed using an Illumina HiSeq X Ten system at Anoroad Biotech Co., Ltd. (Beijing, China) to produce 150-bp-end sequences for the two pools. The raw data were submitted to the Sequence Read Archive (SRA) of NCBI under the accession ID PRJNA1095211. The genome of DH40 served as the reference for calling the SNP variants using the SAMtools software [79]. The SNP-index for each pool was determined by calculating the percentage of reads containing SNPs out of the total number of reads [80]. Subsequently, the △(SNP-index) of the individual loci were computed by subtracting the value of the SNP-index of the R-pool from that of the S-pool, following the methodologies outlined in previous studies [37, 81]. The candidate region was pinpointed through a sliding window approach using a window size of 1 Mb and a step size of 50 kb at 99% level of confidence.

### Candidate gene cloning and expression analysis

We designed primers according to the assembled DH40 genome and cloned the DNA and cDNA sequence of the candidate gene using Phanta® High-Fidelity enzyme Mix P520 (Vazyme, Nanjing, China). The amplified polymerase chain rection (PCR) fragments were sequenced by Sunya Biotech Co., Ltd. (Zhengzhou, China). The sequences were aligned using the MUSCLE webserver (https://www.ebi.ac.uk/Tools/msa/muscle/). The CDS of the resistant DH40 line were deposited in GenBank under the accession PP593248.

For relative expression analysis, the total RNA was extracted using the RNAiso Plus reagent (TaKaRa, Japan), and the first-strand cDNA was synthesized using the TransScript One-Step gDNA Removal and cDNA Synthesis Kit (Trans, Beijing, China). Subsequent quantitative real-time PCR (qRT-PCR), was conducted using the SYBR Premix Ex TaqTM II (TaKaRa, Japan).

### Subcellular localization

The CDSs of *Crr5* devoid of the stop codon were amplified from the CR DH40 line using the Crr5-fulF and Crr5-fulR2 primer pairs (Table S24). These amplified cDNA fragments were then integrated into the modified pBWA(V)HS vector, which features a green fluorescent protein at its C terminus. Subsequently, these recombinant plasmids were introduced by *Agrobacterium*-mediated transformation into the epidermal cells of tobacco leaves. The pNLS-RFP served as the positive control [82]. The resulting fluorescence was visualized using a confocal laser scanning microscope (Nikon C1, Japan).

### GUS assay construct and histochemical staining

The 2.5-kb sequence 5′-upstream of the *Crr5* gene in the resistant DH40 line were amplified using primer pairs Crr5-pro-F and Crr5-pro-R (Table S24). The products were constructed into the binary vector of pCAMBIA1300 for constructing the Crr5Pro::GUS construct, which was introduced into *Arabidopsis* Col-0. The T_2_ plants were utilized for the reporter gene assays. For histochemical staining analysis, transgenetic *Arabidopsis* lines were soaked in 2 mM of 5-bromo-4-chloro-3-indolyl-β-D-glucuronide solution for a period of 12 h. Subsequently, the chlorophyll was removed by treating the stained seedlings using 70% ethanol.

### Cell death assay

The Crr5-fulF and Crr5-fulR primer pairs were used for amplifying the full-length CDS of *Crr5*, while the Crr5-fulF and Crr5-TIR-256R primer pairs were used for amplifying the sequence of N-TIR (Table S24). The PCR products were cloned into pER8, which is an estradiol-inducible expression construct [83], to generate pER8-Crr5 and pER8-Crr5-TIR, respectively. The resultant constructs were expressed transiently in the leaves of tobacco using *Agrobacterium* GV3101. Following 24-h cultivation, 4 μM β-estradiol was sprayed on the areas overlapping the initial infiltration, which was performed by Wang *et al.,* (2023) [84]. Cell death was determined after 48 h of treatment with estradiol, and leaves were photographed. The pER8-WTS [84] was used as the positive control.

### Generation of *Arabidopsis* plants overexpressing *Crr5*

The GV3101 strain of *Agrobacterium tumefaciens* harboring the pBWA(V)HS-p35S-Crr5-GFP construct used for studying the subcellular localization of Crr5 was also transformed into *Arabidopsis* Col-0 using the floral dip technique. The T_1_ seedlings were grown on MS medium using 15 mg/l glufosinate ammonium. Following validating of the positive transformants, a total of three T_2_ transgenetic lines of *Arabidopsis* were selected for subsequent analysis of the phenotypes.

## Supplementary Material

Web_Material_uhae338

## Data Availability

The genome sequence and annotation file of Chinese cabbage DH40 were submitted to the National Genomics Data Center under (accession ID: GWHERQJ00000000) and Figshare ( 10.6084/m9.figshare.25532824.v1). The Hi-C and transcriptome data have been submitted to the National Genomics Data Center (accession ID: PRJCA024893), and the HiFi data are available at NCBI SRA (accession ID: SRR28482076).
