# Supplementary material for: A chromosome-level reference genome facilitates the discovery of clubroot-resistant gene Crr5 in Chinese cabbage
Source: Hortic Res. 2024 Dec 4;12(3):uhae338. doi: 10.1093/hr/uhae338 (PMC11879649; doi:10.1093/hr/uhae338)
Supplement: Web_Material_uhae338 [file web_material_uhae338.zip › Supplemental Figures-20241028.docx]

**Supplemental Figures**

**
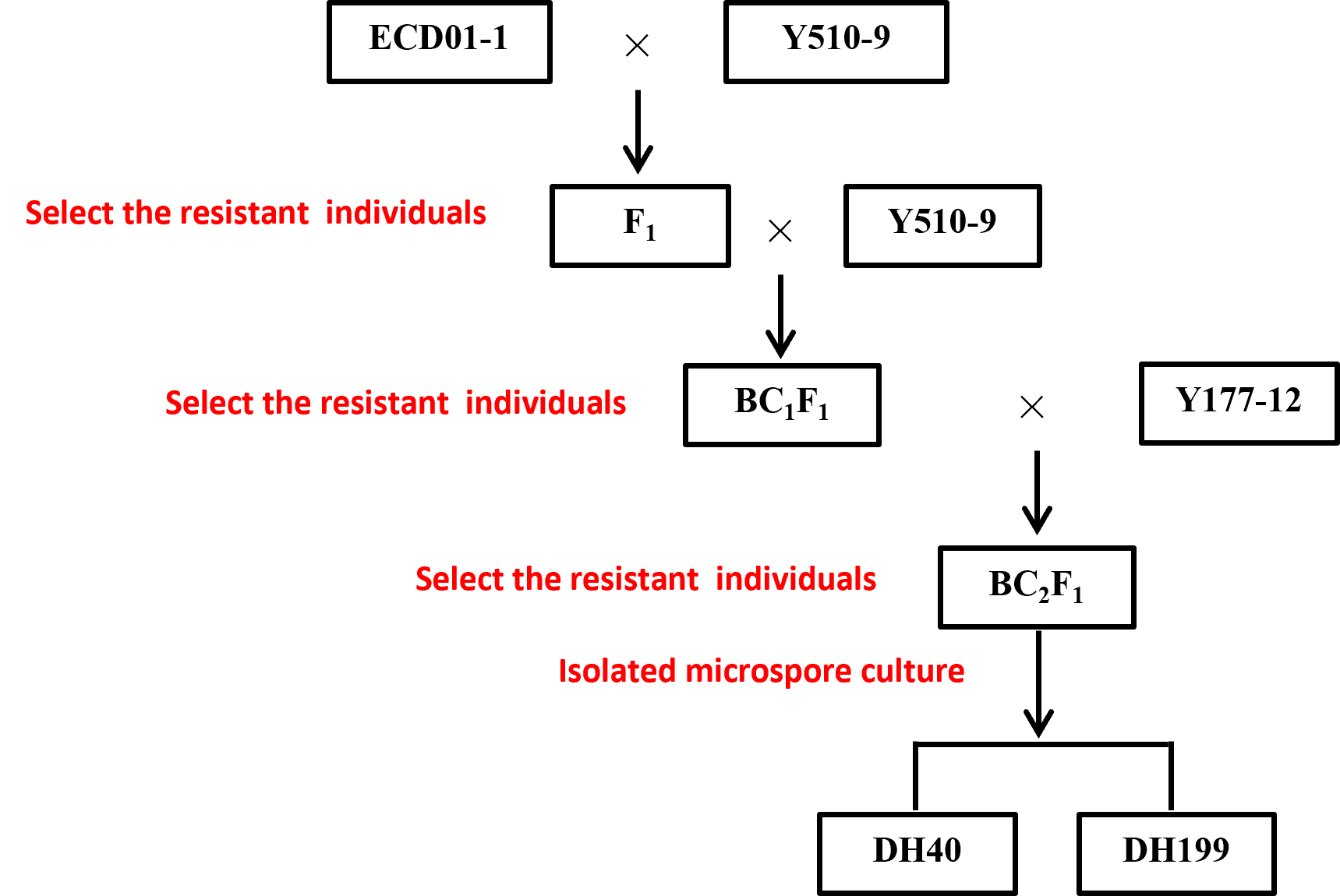
**

**Figure S1.** The pedigree of DH40 and DH199.


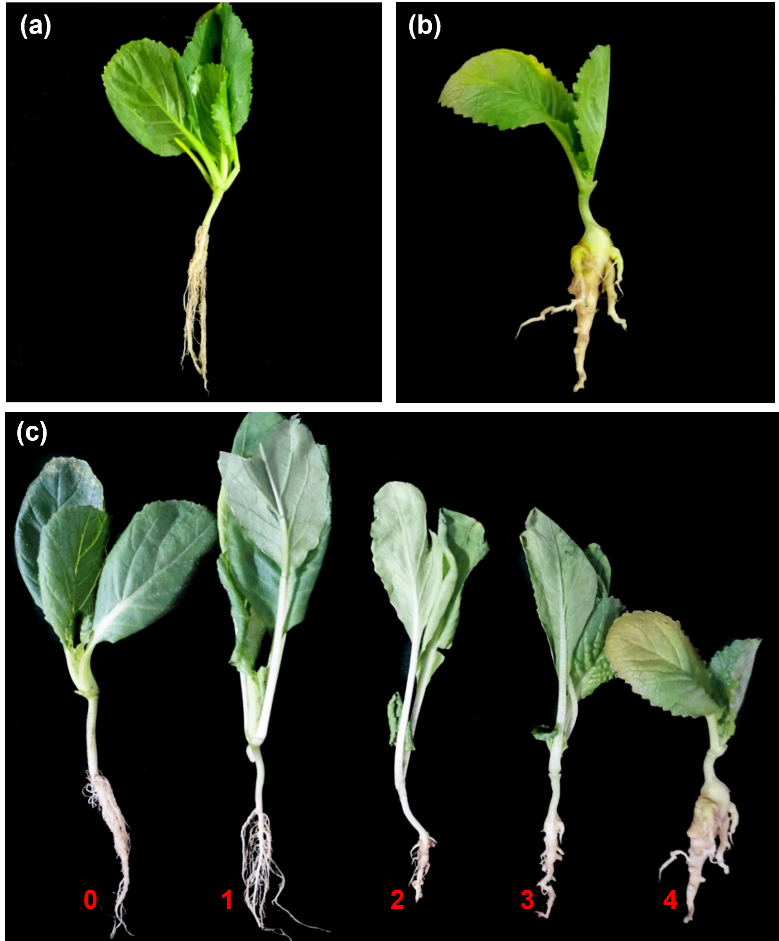


**Figure S2.** Clubroot resistance phenotype of parents and F_2_ population.


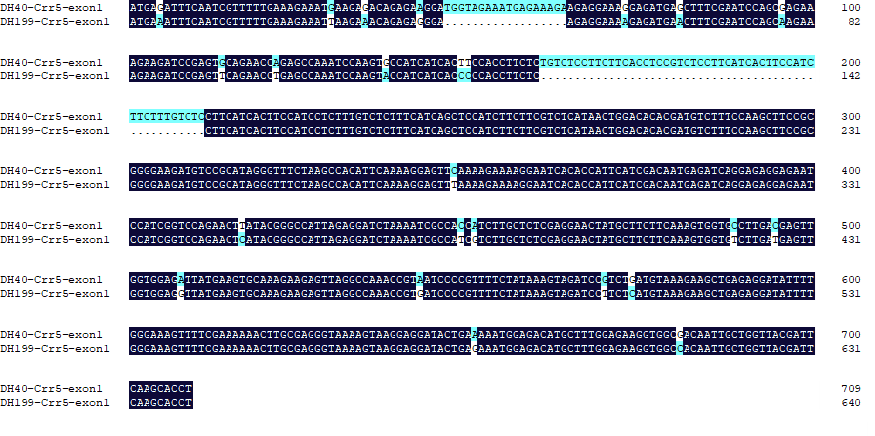


**Figure S3.** Sequence alignment of *Crr5* exon1 between DH40 and DH199.

**Figure S4.** Sequence alignment of *Crr5* intron1 between DH40 and DH199.

**Figure S5.** Sequence alignment of *Crr5* exon2 between DH40 and DH199.


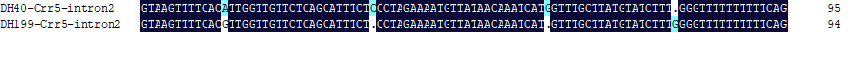


**Figure S6.** Sequence alignment of *Crr5* intron2 between DH40 and DH199.


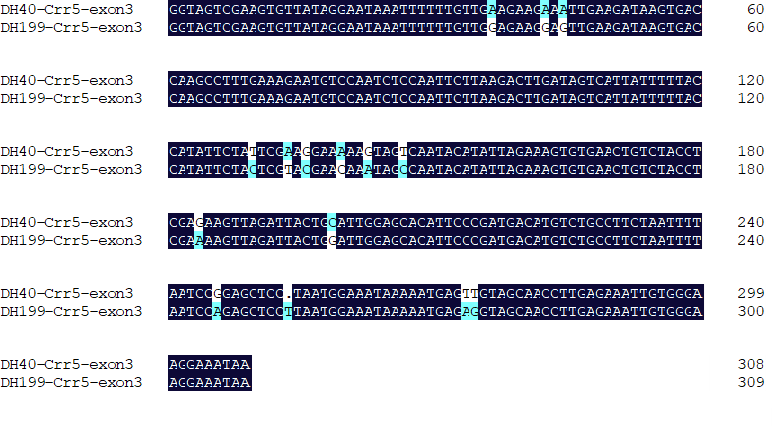


**Figure S7.** Sequence alignment of *Crr5* exon3 between DH40 and DH199.


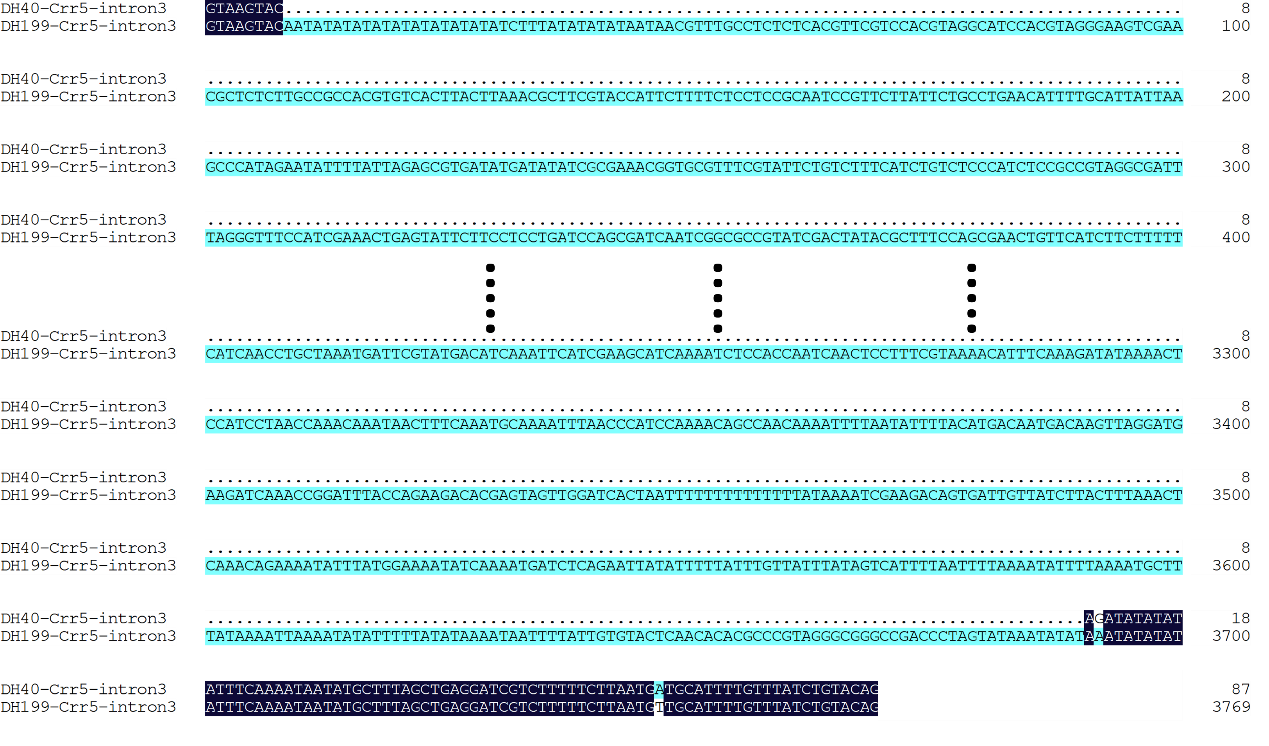


**Figure S8.** Sequence alignment of *Crr5* intron3 between DH40 and DH199.

**Figure S9.** Sequence alignment of *Crr5* exon4 between DH40 and DH199.


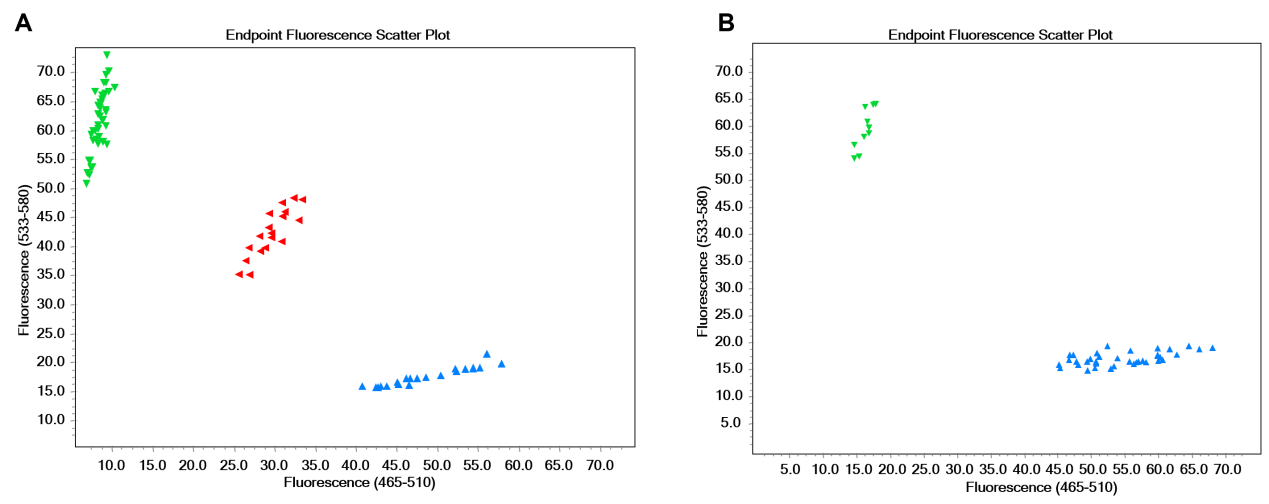


**Figure S10.** Validation of the Crr5-funK1 marker in (DH40×DH199)-F_2_ population (A) and in a natural population (B).


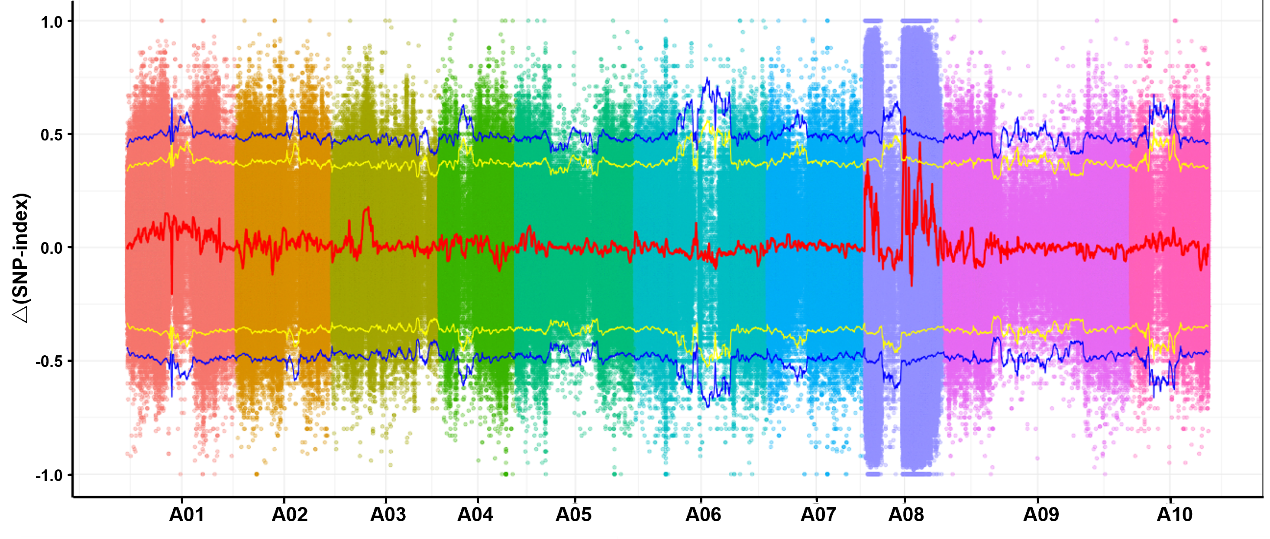


**Figure S11.** BSA-seq analysis for *Crr5* using Chiifu V4.0 as the reference genome.


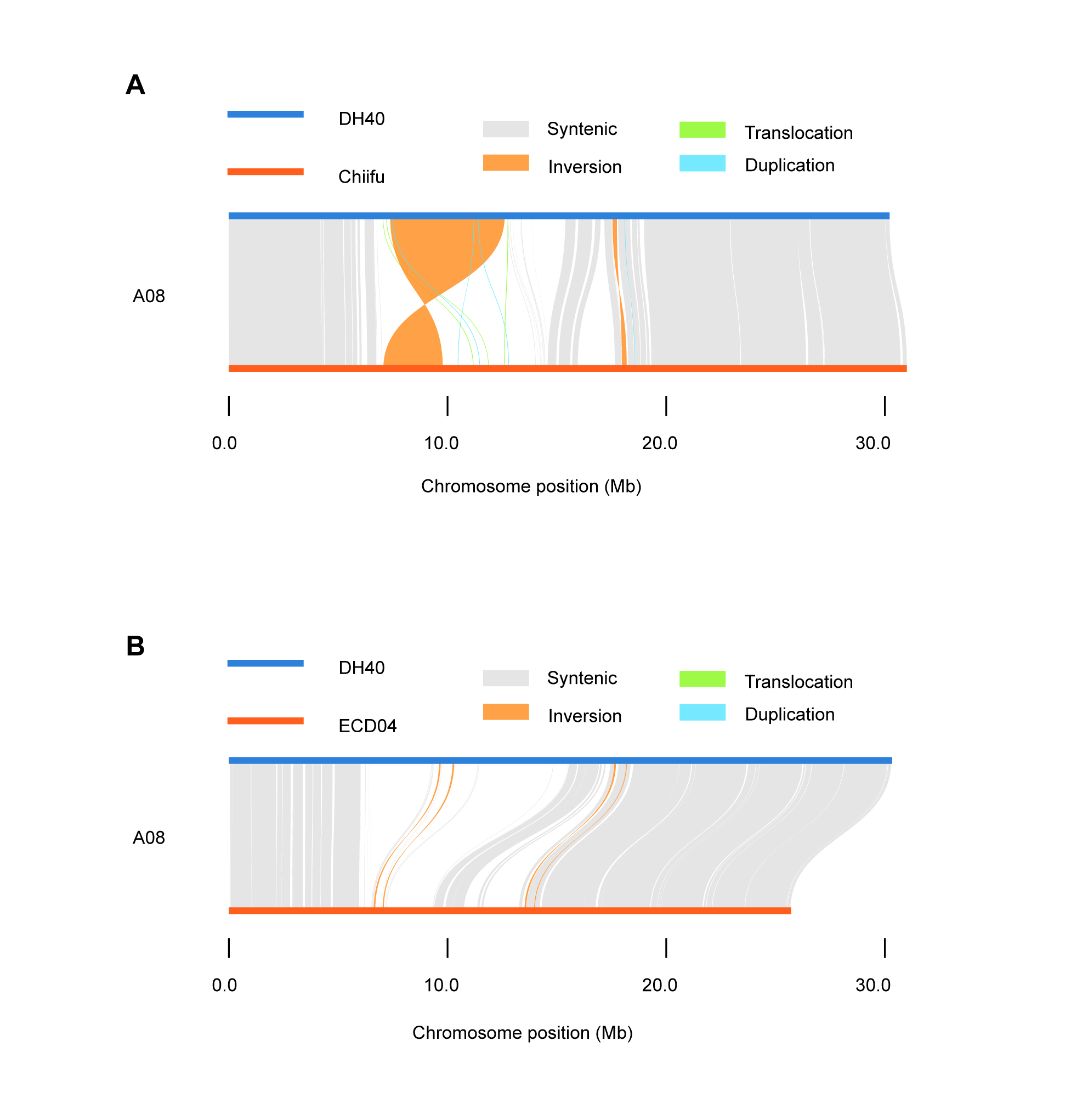


**Figure S12.** Chromosome of A08 comparison of DH40 with Chiifu V4.0 (A) and ECD04 (B).

**Figure S13.** Sequence alignment of CDS of *Crr5* and *Crr1a* .


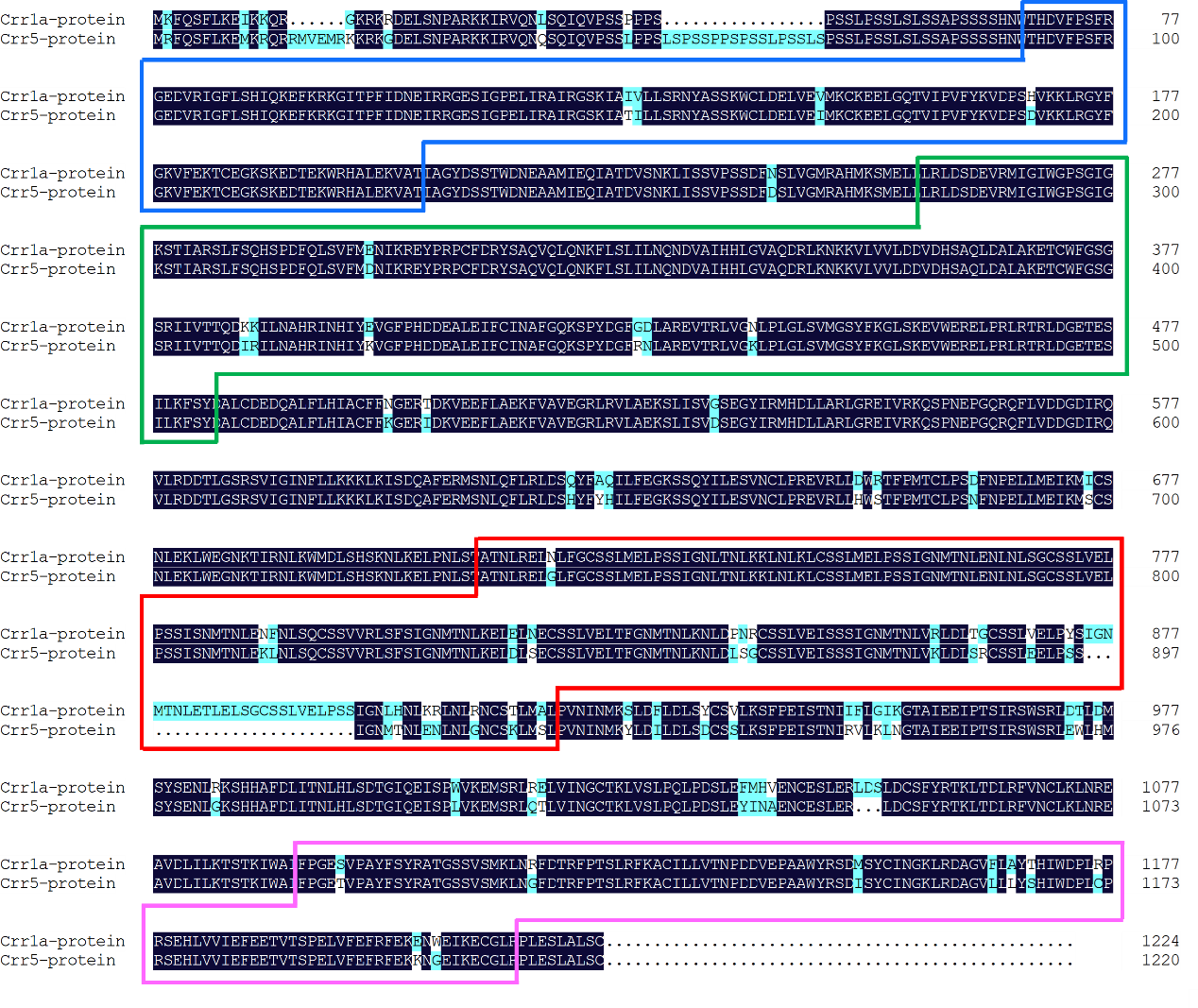


**Figure S14.** Alignment of protein sequence of *Crr5* and *Crr1a* Blue, TIR domain; Green, NB domain; red, LRR domain; Pink, C-JID domain.
